# Integrating Social Determinants of Health With Tobacco Treatment for Individuals With Opioid Use Disorder: Feasibility and Acceptability Study of Delivery Through Text Messaging

**DOI:** 10.2196/36919

**Published:** 2022-09-01

**Authors:** Hasmeena Kathuria, Divya Shankar, Vinson Cobb, Julia Newman, Katia Bulekova, Scott Werntz, Belinda Borrelli

**Affiliations:** 1 The Pulmonary Center Boston University School of Medicine Boston, MA United States; 2 Boston University Boston, MA United States; 3 Agile Health Lincolnshire, IL United States; 4 Henry M Goldman School of Dental Medicine Boston University Boston, MA United States

**Keywords:** text message, smoking cessation, opioid use disorder, tobacco dependence, tobacco treatment interventions, mobile phone

## Abstract

**Background:**

Individuals with opioid use disorder (OUD) have a high prevalence of smoking and frequently experience unmet social determinants of health (SDOH), which may be barriers to smoking cessation. Hospitalization is an opportunity to encourage smoking cessation. Unfortunately, many clinicians do not provide tobacco treatment to support the maintenance of cessation achieved during hospitalization. Interventions are required to support these high-risk individuals after hospital discharge.

**Objective:**

This study aimed to test the feasibility and acceptability of a 28-day SMS text messaging program tailored to individuals with OUD, which provides smoking cessation support and addresses unmet SDOH needs.

**Methods:**

From July to December 2019, we enrolled 25 individuals who were hospitalized with tobacco dependence and OUD at our large safety net hospital. The SMS text messaging program was initiated during hospitalization and continued for 28 days. Participants were enrolled in either the *ready to quit within 30 days* or the *not ready to quit within 30 days* program based on their readiness to quit. Automated SMS text messages were sent twice daily for 4 weeks. The topics included health and cost benefits of quitting, both general and opioid specific (16 messages); managing mood and stress (8 messages); motivation, coping strategies, and encouragement (18 messages); addressing medication misconceptions (5 messages); links to resources to address substance use (2 messages providing links to the Massachusetts Substance Use Helpline and Boston Medical Center resources), tobacco dependence (1 message providing a link to the Massachusetts Quitline), and unmet SDOH needs (6 messages assessing SDOH needs with links to resources if unmet SDOH needs were identified). Questionnaires and interviews were conducted at baseline and at 2 and 4 weeks after enrollment.

**Results:**

The participants were 56% (14/25) female, 36% (9/25) African American, 92% (23/25) unemployed, and 96% (24/25) Medicaid insured. Approximately 84% (21/25) activated the program, and none of the participants unsubscribed. Approximately 57% (12/21) completed either the 2- or 4-week questionnaires. Program satisfaction was high (overall mean 6.7, SD 0.8, range 1-7). Many perceived that the SMS text messaging program provided social support, companionship, and motivation to stop smoking. Messages about the health benefits of quitting were well received, whereas messages on how quitting cigarettes may prevent relapse from other substances had mixed views, highlighting the importance of tailoring interventions to patient preferences.

**Conclusions:**

SMS text messaging to promote smoking cessation and address SDOH needs may be an effective tool for improving quit rates and health outcomes in individuals with tobacco dependence and OUD. Our study adds to the growing body of evidence that SMS text messaging approaches are feasible and acceptable for providing tobacco treatment to all individuals who smoke, even among low-income populations who have OUD and are not ready to quit.

## Introduction

Individuals with opioid use disorder (OUD) smoke at rates as high as 83% to 97% [1,2]. Co-occurring tobacco and opioid use leads to high morbidity and mortality [3,4]. Smoking cessation improves tobacco health-related outcomes and could increase long-term abstinence from opioids [5,6]. A meta-analysis of 24 studies showed a positive impact of smoking cessation on substance use disorder outcomes; both tobacco treatment and smoking cessation either reduced or had no effect on other drug use [7].

Although individuals with OUD desire assistance with smoking cessation [8], tobacco treatment is infrequently offered [9-11]. Hospitalization is an opportunity to encourage smoking cessation [12-16]. We previously found a high acceptance of inpatient tobacco counseling among individuals who were hospitalized with OUD [17]. Unfortunately, many clinicians do not provide tobacco treatment to support the maintenance of cessation achieved during hospitalization [18]. Interventions are required to support these high-risk individuals after hospital discharge.

Individuals who smoke are 1.5 to 2 times more likely to quit smoking when enrolled in SMS text messaging programs for smoking cessation [19-21]. SMS text messaging is highly disseminative: mobile phone ownership is near universal; SMS text messaging is highly prevalent across race, education, and income, and >85% of individuals who smoke send and receive SMS text messages regularly [22-24]. Although studies have not assessed the efficacy of SMS text messaging for smoking cessation in individuals with OUD, they have shown a moderately high reach in Medicaid populations [25].

There is nearly universal agreement in scientific and public health communities that social determinants of health (SDOH)—the social circumstances in which people are born, grow, live, work, and age—influence access to resources and opportunities that affect health [26,27]. SDOH has a far greater impact on health outcomes than medical interventions [28]. For example, cross-sectional and longitudinal studies show that food insecurity is independently associated with an increased likelihood of smoking cigarettes, likely resulting from a combination of physiological factors, including stress, anxiety, and depression [29-31]. Thus, screening for and addressing unmet SDOH needs through policies and interventions may mitigate these factors and improve smoking cessation outcomes.

Many individuals with OUD also experience unmet SDOH needs (eg, transportation issues and food and housing insecurity) [32,33], which may be barriers to smoking cessation. However, studies have not systematically screened for unmet SDOH needs or provided referrals to address these needs in this population. Given their high smoking rates, tobacco-related comorbidities, lack of access to treatment, and inclusion in tobacco treatment trials, an integrative intervention combining tobacco treatment with SDOH assessment and referral may improve smoking cessation among patients with OUD. We sought to iteratively develop and deploy an SMS text messaging program tailored to those with OUD, which provides smoking cessation support and resources to address SDOH. We report the results of a pilot feasibility and acceptability study of an SMS text messaging program initiated during hospitalization and continued for 28 days.

## Methods

### Recruitment and Enrollment

From July to December 2019, we enrolled 25 individuals who were hospitalized with tobacco dependence and OUD at the Boston Medical Center (BMC), the largest safety net hospital in New England. We identified participants from a list of individuals who were hospitalized, who triggered consultation with the Tobacco Treatment Consult service based on current smoking status in the electronic health record [34], and who had an International Classification of Diseases, 10th Revision diagnosis of OUD by chart review. Eligible participants were (1) aged ≥18 years, (2) hospitalized at the BMC, (3) able to speak and read English, (4) currently smoking cigarettes, (5) diagnosed with OUD, (6) mobile phone owners with an unlimited SMS text messaging plan, (7) in agreement to receive SMS text messages for 1 month, (8) not participating in other SMS text messaging or tobacco treatment programs, (9) able to receive a test SMS text message, and (10) able to provide informed consent. The participants were excluded if they were cognitively impaired.

A total of 96 participants met the screening criteria by electronic health record review (individuals listed as current for smoking status and OUD), of whom 42 (44%) were ineligible by face-to-face screening (n=34, 81% did not have unlimited SMS text messaging or had no phone at the time of hospitalization; n=3, 7% had stopped smoking; n=1, 2% did not have OUD; and n=4, 10% could not provide consent), 2 (2%) were unavailable, and 27 (28%) declined or were not interested in learning about the study.

### Ethics Approval

Following the provision of study information, 25 individuals agreed, provided informed consent, and were enrolled. Participants were compensated up to US $50 for participation: US $10 for completing the baseline survey and interview, US $15 for completing a 2-week follow-up survey and interview, and US $25 for completing a 4-week follow-up survey and interview. This study was approved by our institutional review board (protocol number H-38709).

### Structure of Program

The SMS text messaging program lasted 28 days, with the first day of SMS text messages sent during hospitalization. Participants were sent 2 intervention SMS text messages daily (9 AM and 5 PM) in addition to weekly SMS text message assessments (see the *Measures* section). There were 2 tracks: one for individuals *ready to quit within 30 days* and the other for individuals *not ready to quit within 30 days*, as assessed by their answer to an introductory SMS text message assessment. The program had bidirectional or 2-way SMS text messaging capabilities; for example, participants could text a keyword (eg, *CRAVE*) to receive strategies and tips.

The SMS text messages were fully automated. All incoming SMS text messages were monitored and, if needed, responded to in real time by a team member via a password-protected dashboard interface if the system did not recognize an SMS text message and could not produce an automated response. SMS text messages were delivered by Agile Health, Inc, and their system is Health Insurance Portability and Accountability Act compliant. All the data managed by Agile Health and its message delivery partners were encrypted in transit and at rest. All user interactions, comprising solicited and unsolicited SMS text messages were recorded, including “STOP” (the standard keyword for unsubscribing). Agile Health, Inc notified the research team members of the urgent unsolicited SMS text messages sent to the server. All SMS text messages sent by the participant to, and responded by, the system were reviewed by the research team weekly.

### Program Content

The topics of the SMS text messages included (1) health and cost benefits of quitting, both general and opioid specific (16 messages); (2) managing mood and stress (8 messages); (3) motivation, coping strategies, and encouragement (18 messages); (4) addressing medication misconceptions (5 messages); (5) links to resources to address substance use (2 messages providing links to the Massachusetts Substance Use Helpline and BMC resources), tobacco dependence (1 message providing link to Massachusetts Quitline), and resources for unmet SDOH needs (6 messages assessing SDOH needs with links to resources if SDOH needs were unmet). Messages were obtained from three sources: (1) the National Cancer Institute’s Smokefree TXT [35], (2) content adapted from prior work by Borrelli et al [36], and (3) novel messages developed by the study team.

**Figure 1:**
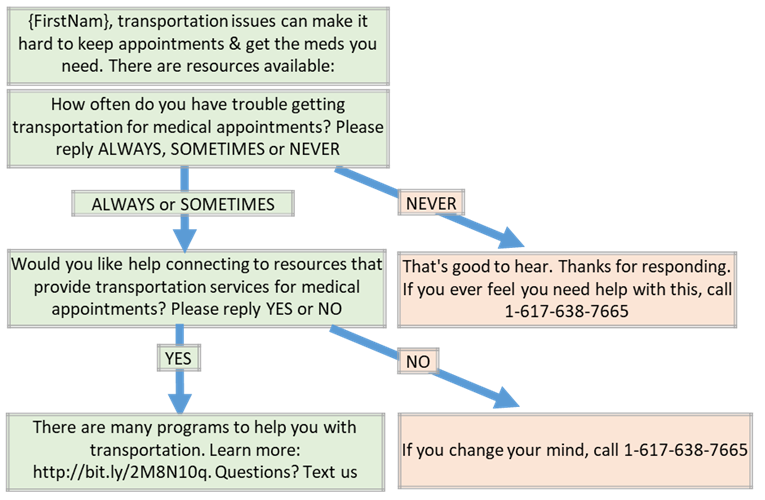
Example algorithm for assessment of unmet social determinants of health needs and for provision of resources to address the need.

Assessments and links to resources were provided for the following 6 SDOH needs: difficulty with transportation to medical appointments, inability to pay for medications, risk of becoming homeless, food insecurity, trouble paying for heat or electricity, and the likelihood of needing to look for a job (Multimedia Appendix 1). Individuals were considered to have an unmet SDOH need if they answered either “ALWAYS” or “SOMETIMES” to an SMS text message assessing for the SDOH need (example SMS text message: “How often do you have trouble getting transportation for medical appointments? Please reply ALWAYS, SOMETIMES, or NEVER”). Participants were then sent a link to resources if they answered “YES” to a text assessing their desire for help with that need (eg, “Would you like help connecting to resources that provide transportation services for medical appointments? Please reply YES or NO”). Figure 1 outlines the algorithm for the provision of resources.

Assessments for the 6 SDOH needs were adapted from the validated Tool for Health and Resilience In Vulnerable Environments (THRIVE) screening tool and accompanying referral guide [37]; the THRIVE screening tool asks about 8 SDOH domains (housing, food, affording medications, transportation, utilities, caregiving, education, and employment) selected based on their impact on health and available services in the community. The THRIVE referral guide is a web-based directory of resources with contact information for community services to meet the SDOH needs. The need for caregiving and education were not assessed to reduce participant burden and also as we believed that addressing these SDOH needs would require a more nuanced discussion with an advocate or community health worker.

Several SMS text messages (3-5 messages per week) were customized based on the readiness to quit. In the *ready to quit* track, an SMS text message on coping strategies was “Cravings will get weaker and less frequent with every day that you don’t smoke.” In the *not ready to quit* track, a parallel message was “Don’t let cravings get in the way of deciding to quit. There are good meds to help with cravings. Cravings get weaker with each passing day.” Messages in the *not ready to quit* track were directed toward developing participants’ personal reasons for change and increasing their motivation and self-efficacy to stop smoking. Examples of such messages are “Thought: What is the best result you can imagine if you quit smoking? Imagine all the ways your life would change. How would you spend the extra money? How would you feel? Who would you spend time with? Where would you spend your time?” and “Thought for the day: Fall down 7 times, get up 8. The key to success is to persist even if you have previously failed.” Messages in the *ready to quit* track provided encouragement; an example was “Stay positive. Your journey to a smokefree life may be a struggle, but looking back it will be well worth it.”

### Measures

The study staff administered baseline questionnaires and interviews in person at the time of hospitalization and conducted 2- and 4-week questionnaires and interviews by telephone. Questionnaires followed by qualitative interviews were administered at the same encounter.

#### Participant Characteristics

Baseline demographics, use of substances, and comorbid mental health disorders were collected at the time of enrollment. Mobile technology use and smoking characteristics were assessed, including the level of cigarette dependence measured using the Fagerstrom Test for Cigarette Dependence [38,39]. Scores range from 0 to 10, with higher scores indicating more intense physical dependence on nicotine. No dependence corresponds to a score of 0, low dependence to a score of 1 or 2, low to moderate dependence to a score of 3 or 4, moderate dependence to a score of 5 to 7, and high dependence to a score of 8 to 10. We assessed psychosocial characteristics by assessing responses to the following question: “Have you ever been diagnosed with any of the following (check all that apply)? Answer choices: Depression; Anxiety; Bipolar Disorder; Manic-Depressive disorder; None of the above; Prefer not to answer.”

#### Program Engagement and Interactions With Program

Engagement was assessed through participants’ interactions with the program. We calculated the *total* *response rate* by computing the number of participant-submitted responses to SMS text messages in which a response was expected and dividing this by the number of solicited responses. The number of *unsolicited* SMS text messages (messages sent by users where a response was not expected, such as “Thanks you guys are a big help”) was an additional index of engagement.

#### Program Satisfaction

Program satisfaction was measured via 2- and 4-week questionnaires, using several indices of satisfaction. The “share-worthiness” of the SMS text messages was assessed by asking whether participants showed the SMS text messages to others (response: yes/no) and the extent to which they believed the SMS text messages would be helpful to family and friends (range 1=not at all helpful to 7=very helpful). The perceived quality of the SMS text messages was measured using 2 items from the Mobile Application Rating Scale [40]: one using star ratings (1 star=one of the worst SMS text message programs to 5 stars=one of the best SMS text message programs) and the other assessing how much longer they would have liked to receive the SMS text messages. Satisfaction with the program components and overall program satisfaction were assessed using 9 items (range 1=not satisfied at all to 7=very much satisfied). The likeability of program components was assessed using 7 items (range 1=did not like it at all to 7=liked it very much). The full list of items used to assess program satisfaction is presented in the *Results* section.

Satisfaction was additionally measured by eliciting responses on the helpfulness of the content of 8 specific SMS text messages (2 times per week, an intervention message was followed by an assessment SMS text message asking participants the following: “How helpful did you find this text? 3=Very helpful, 2=Neutral, or 1=Not helpful”). The 8 specific SMS text messages that were assessed for the helpfulness of the content are presented in the *Results* section.

#### Perceived Impact of the Program

Weekly SMS text messages assessed the degree to which the program was helpful in motivating smoking cessation (range 1=not helpful to 5=very helpful) [40]. The 2- and 4-week questionnaires assessed the perceived impact of the program on (1) motivation to stop smoking, (2) belief that stopping opioids and smoking cessation can occur simultaneously, and (3) knowledge of health risks from smoking (range 1=not at all to 7=very much).

#### Qualitative Assessment of Feasibility, Acceptability, and Satisfaction

We qualitatively measured feasibility and acceptability at 2 and 4 weeks using semistructured interview guides. The guide assessed the participants’ (1) perceived impact in motivating smoking cessation, (2) experiences with the program, (3) preference of content, and (4) suggestions for improvement.

### Data Analyses

Basic descriptive statistics were calculated using SPSS (version 18; IBM Corp) to summarize the quantitative responses. For qualitative interviews, we used inductive content analysis to analyze transcripts and performed unstructured coding of the transcripts to identify themes. A total of 2 members developed a codebook, independently reviewed all transcripts, and added codes until the team reached a consensus. We finalized the conceptual categories, grouped themes in each category, and identified quotes that best highlighted the themes. The interviews were audio recorded and transcribed verbatim.

## Results

### Quantitative Analyses

#### Participant Characteristics

The participants were 56% (14/25) female, 36% (9/25) African American, 20% (5/25) Hispanic, 92% (23/25) unemployed, and 96% (24/25) Medicaid insured. The mean age was 45.8 (SD 11, range 28-63) years. Of the 25 participants, 12 (48%) reported current opioid use, and 12 (48%) were receiving medication-assisted treatment for OUD. Participants smoked for an average of 26.2 (SD 12, range 3-48) years. The Fagerstrom Test for Cigarette Dependence scores averaged 5.1 (SD 2), suggesting moderate nicotine dependence (Table 1). Approximately 44% (11/25) of the participants were ready to stop smoking within 30 days (Table 1).

**Table 1:** Characteristics of participants (N=25).

Baseline characteristics				All participants		Ready to quit track (n=14)		Not ready to quit track (n=11)
Age (years), mean (SD)				45.8 (11)		50.8 (9.5)		39.6 (8.8)
Sex (female), n (%)				14 (56)		7 (50)		7 (64)
**Race, n (%)**								
	Black or African American		9 (36)		4 (29)		5 (45)	
	White		13 (52)		8 (57)		5 (45)	
	Other		3 (12)		2 (14)		1 (9)	
Hispanic ethnicity, n (%)				5 (20)		3 (21)		2 (18)
Medicaid insurance, n (%)				24 (96)		13 (93)		11 (100)
**Education, n (%)**								
	Less than high school		7 (28)		4 (29)		3 (27)	
	High school or General Educational Development		11 (44)		6 (43)		5 (45)	
	More than high school		7 (28)		4 (29)		3 (27)	
**Housing situation, n (%)**								
	At risk of homelessness		3 (12)		2 (14)		1 (9)	
	Experiencing homelessness		12 (48)		5 (36)		7 (64)	
Divorced or separated, widowed, or never married, n (%)				20 (80)		12 (86)		8 (73)
Unemployed, n (%)				23 (92)		13 (93)		10 (91)
**Yearly household income before taxes (US $), n (%)**								
	0-14,999		17 (68)		9 (64)		8 (73)	
	>15,000		4 (16)		3 (21)		1 (9)	
	Prefer not to answer or do not know		4 (16)		2 (14)		2 (18)	
Depression or anxiety, n (%)				19 (76)		9 (64)		10 (91)
**Current use of substances, n (%)**								
	Alcohol (≥5 for men and ≥4 for women in 1 day)		5 (25)		3 (21)		1 (9)	
	Cocaine		13 (52)		4 (29)		9 (82)	
	Opiates		12 (48)		7 (50)		5 (45)	
	Marijuana		12 (48)		7 (50)		5 (45)	
	Prescription drugs (not prescribed)		6 (24)		2 (14)		4 (36)	
	Methamphetamines		4 (16)		1 (7)		3 (27)	
**Smoking characteristics**								
	Years smoked, mean (SD)		26.2 (12)		27.9 (13.4)		24.1 (9.4)	
	Smokes daily, n (%)		25 (100)		14 (100)		11 (100)	
	**Importance of quitting smoking, n (%)**							
		Very important or important	17 (68)		12 (86)		5 (45)	
		Neutral	4 (16)		1 (7)		3 (27)	
		Low importance or not important	4 (16)		1 (7)		3 (27)	
	**Motivation to quit smoking, n (%)**							
		Very motivated or motivated	16 (64)		12 (86)		4 (36)	
		Somewhat or slightly motivated	7 (28)		2 (14)		5 (45)	
		Not at all motivated	2 (8)		0 (0)		2 (18)	
	Fagerstrom score, mean (SD)		5.1 (2)		5.6 (2.4)		4.6 (1.4)	
	Dual use of cigarettes and e-cigarettes, n (%)		5 (25)		0 (0)		5 (45)	
**Mobile technology, n (%)**								
	Smartphone ownership		23 (92)		13 (93)		10 (91)	
	**SMS text messages sent per day per week, n (%)**							
		2-9 per day	7 (28)		5 (36)		2 (18)	
		>10 per day	17 (68)		9 (64)		8 (73)	
		2-6 per week	1 (4)		0 (0)		1 (9)	

#### Engagement and Interactions With the Text Messaging Program

While in the hospital, and after obtaining informed consent, participants were asked via SMS text message whether they would like to begin (*activate*) the program. Of the 25 participants, 21 (84%; n=11, 52% *ready to quit*, and n=10, 48% *not ready to quit*) responded with *yes*. Of the 25 participants, 2 (8%) did not activate the program and expressed concern about not having access to a phone after discharge (eg, attending a rehabilitation program and concern about minutes); the other 2 (8%) were not available to assess reasons for not activating. Of the 21 participants, 13 (62%; n=5, 38% *ready to quit*, and n=8, 62% *not ready to quit*) submitted at least one response to the helpfulness assessments of 8 intervention SMS text messages (55/168, 32.7% response rate); 13 (62%; n=6, 46% *ready to quit*, and n=7, 54% *not ready to quit*) submitted at least one response to the SMS text messages assessing SDOH needs (44/147, 29.9% response rate); 15 (71%; n=7, 47% *ready to quit*, and n=8, 53% *not ready to quit*) responded to at least one weekly text SMS message assessment regarding the degree to which the program was helpful in motivating cessation (35/84, 42% response rate); and 14 (67%; n=7, 50% *ready to quit*, n=8, 57% *not ready to quit*) sent at least one unsolicited SMS text message for a total of 143 unsolicited messages. None of the participants unsubscribed.

#### Perceptions of Program

Of the 21 participants, 12 (57%; n=7, 58% *ready to quit*, and n=5, 42% *not ready to quit*) completed either the 2- or 4-week questionnaires:6 (50%; n=3, 50% *ready to quit*, and n=3, 50% *not ready to quit*) completed both questionnaires (2- and 4-week responses were similar; only 4-week responses were analyzed); 4 (33%; n=2, 50% *ready to quit*, and n=2, 50% *not ready to quit*) completed only the 2-week questionnaire; and 2 (17%; n=1, 50% *ready to quit*, and n=1, 50% *not ready to quit*) completed only the 4-week questionnaire.

##### Program Satisfaction

Of the 12 participants, 10 (83%; n=6, 60% *ready to quit*, and n=4, 40% *not ready to quit*) rated the program ≥4 stars, and 2 (16%; n=1, 50% *ready to quit*, and n=1, 50% *not ready to quit*) rated the program 3 stars. Of the 12 participants, 4 (33%; n=3, 75% *ready to quit*, and n=1, 25% *not ready to quit*) wanted the program to last up to 2 months longer, and 8 (67%; n=4, 50% *ready to quit*, n=4, 50% *not ready to quit*) indicated that they wanted the program to last ≥3 months longer. None of the participants thought that the program had interfered with their schedules. Of the 12 participants, 9 (75%; n=5, 56% *ready to quit*, and n=4, 44% *not ready to quit*) shared the SMS text messages with others. Participants believed that the SMS text messages would be helpful to family and friends (mean 5.4, SD 1.1, range 1-7; *ready to quit* mean 5.1, SD 1; *not ready to quit* mean 5.6, SD 1). Of the 12 participants, 11 (92%; n=7, 64% *ready to quit*, and n=4, 36% *not ready to quit*) were very likely or likely to recommend the program to others, and 1 (8%) person in the *not ready to quit* track was somewhat likely to recommend the program to others.

Overall, program satisfaction was high (overall mean 6.7, SD 0.8, range 1-7; *ready to quit* mean 7, SD 0; *not ready to quit* mean 6.2, SD 0.98). Participants reported that the content was trustworthy (mean 6.5, SD 0.8). Most liked the frequency (mean 5.8, SD 1.8, range 1-7) and timing (mean 5.9, SD 1.2, range 1-7) of the SMS text messages (Table 2).

**Table 2:** Questionnaire responses on likability, satisfaction, and perceived impact of the SMS text messaging program (N=12).

Items			Overall, mean (SD)		*Ready to quit* track (n=7), mean (SD)		*Not ready to quit* track (n=5), mean (SD)
**Likeability scale items^a^**							
	Having to respond to SMS text message questions	5.4 (2.2)		5 (2.6)		6 (1.3)	
	The degree to which the program was interesting	5.3 (1)		5.1 (1)		5.6 (1)	
	The degree to which the program was useful	5.8 (1.3)		5.7 (1.2)		6 (1.5)	
	The degree to which the program was engaging	5.7 (1.4)		6 (1.3)		5.2 (1.3)	
	The degree to which the program was boring	1.8 (1.1)		1.9 (1.4)		1.8 (0.7)	
	The frequency with which texts were delivered	5.8 (1.7)		5.9 (2.1)		5.8 (1.2)	
	The time of the day that texts were received	5.9 (1.2)		6.3 (1.2)		5.4 (1)	
**Program satisfaction scale items^b^**							
	Overall satisfaction with the program	6.7 (0.8)		7 (0)		6.2 (1)	
	Receipt of support when needed	5.9 (1.1)		5.6 (1.2)		6.4 (0.8)	
	The amount of information in the SMS text messages	5.9 (1.2)		6.1 (1.1)		5.6 (1.2)	
	The quality of the information in the SMS text messages	5.8 (1.3)		5.9 (1.4)		5.6 (1.2)	
	Relevancy of program for self	5.4 (1.8)		5.4 (2.1)		5.4 (1.5)	
	The trustworthiness of the information	6.5 (0.8)		6.7 (0.7)		6.2 (0.75)	
	The level of program customization	5.6 (1.3)		5.6 (1.2)		5.6 (1.5)	
	The degree to which the SMS text messages were well written	6.8 (0.6)		6.9 (0.3)		6.6 (0.8)	
	The degree to which the SMS text messages were easy to integrate into routine	6.2 (1.1)		6.1 (1.4)		6.2 (0.7)	
**Perceived impact of the program on motivation to stop smoking, beliefs, and overall knowledge of health risk^c^**							
	The degree to which the program motivated you to quit smoking	6.2 (1.2)		6.7 (0.5)		5.4 (1)	
	Belief that opioids and smoking cessation can occur at the same time	4.5 (2.5)		4.7 (2.7)		4.4 (2.3)	
	Overall knowledge about the health risk of smoking	5.6 (1.9)		6.1 (1.4)		4.8 (2.2)	

^a^Range: 1=did not like it at all to 7=liked it very much*.*

^b^Range: 1=not satisfied at all to 7=very much satisfied*.*

^c^Range: 1=not helpful to 7=very helpful in motivating smoking cessation.

##### Perception of Program Content

Of the 12 participants, 9 (75%; n=5,56% *ready to quit*, and n=4, 44% *not ready to quit*) rated SDOH the SMS text messages (eg, where to find food pantries) *helpful*, and 10 (83%; n=6, 60% *ready to quit*, and n=4, 40% *not ready to quit*) rated the SMS text messages on managing mood and stress as helpful. All 12 participants rated the SMS text messages on resources for quitting smoking as helpful, and 11 (92%; n=6, 55% *ready to quit*, and n=5, 45% *not ready to quit*) rated the SMS text messages about where to find help for other substances as helpful. The overall response rates for the SMS text message assessments on helpfulness (3=very helpful, 2=neutral, and 1=not helpful) of the 8 specific intervention messages were low (response rates ranged from 23.8% to 57.1%; Table 3). Participants in both tracks gave high ratings for messages about managing mood and stress, addressing medication misconceptions, and increasing their motivation to quit. Participants were neutral regarding messages about the benefits of quitting smoking on the use of other substances (Table 3).

**Table 3:** Response rates and ratings of 8 specific SMS text messages that were assessed for the helpfulness of content (N=21)^a^.

Rating	Text (specific SMS text messages assessed)	Total		Ready to quit track			Not ready to quit track	
		Response rate (%)	Rating, mean (SD)	Response rate (n=11; %)	Rating, mean (SD)	Response rate (n=10; %)		Rating, mean (SD)
Helpful 1: managing mood and stress	“Stress Tip: Talk about your problems! This lowers stress and gives new perspectives. Holding it in could affect your health and wellness”	57.1	2.7 (0.5)	45.5	2.6 (0.5)	70		2.7 (0.5)
Helpful 2: tips for cravings	“Whenever you want a cig, try the four D’s: Delay, Deep breathe, Drink water, Do something to take your mind off smoking”	28.6	2.5 (0.5)	36.3	2.5 (0.5)	20		2.5 (0.5)
Helpful 3: addressing medication mistrust	“MYTH: Chantix/Wellbutrin will make me feel depressed. FACT: Research shows no evidence that these meds increase risk of suicide & depression”	28.7	2.7 (0.5)	18.2	2.5 (0.5)	40		2.7 (0.5)
Helpful 4: motivating to quit	“Thought for the day: What would get easier in your life if you didn’t smoke? No more worrying about finding money to buy cigarettes and where you can smoke. Less worry about your health. What else would get better?”	42.9	2.6 (0.7)	36.3	3 (0)	50		2.2 (0.7)
Helpful 5: benefits of quitting (opiate specific)	“Smokers in substance use treatment are more likely to die from smoking-related disease compared to complications of their current drug use”	38.1	2.1 (0.8)	36.3	2.5 (0.9)	40		1.8 (0.4)
Helpful 6: motivating to quit	“It might seem like you are giving up a lot when you stop smoking, try to think about all you are gaining”	28.6	2.8 (0.4)	27.3	3 (0)	30		2.7 (0.5)
Helpful 7: benefits of quitting (opiate specific)	“MYTH: Quitting cigarettes could negatively impact recovery. FACT: Smoking cessation may promote recovery in patients who use opioids”	23.8	2.4 (0.5)	27.3	2.7 (0.5)	20		2 (0)
Helpful 8: managing mood and stress	“What pleasure do you get from smoking? Find healthier alternatives in your life that can bring you these same feelings”	33.3	2.9 (0.3)	45.5	2.8 (0.4)	20		3 (0)

^a^Ratings: 3=very helpful, 2=neutral, and 1=not helpful.

##### Response Rates of Assessments on SDOH Needs

The response rates for SMS text messages assessing SDOH needs varied. All 13 individuals who responded had at least one unmet SDOH need: 5 (38%) had 1 unmet need, 3 (23%) had 2 unmet needs, 1 (8%) had 3 unmet needs, and 4 (31%) had 4 unmet needs. Responses for SDOH needs were as follows: trouble getting transportation for medical appointments (8/13, 62%; n=5, 63% always; n=2, 25% sometimes; and n=1, 13% never), trouble paying for medications (10/13, 77%; n=1, 10% always; n=4, 40% sometimes; and n=5, 50% never), risk of becoming homeless (5/13, 38%; n=3, 60% high; n=1, 20% medium; and n=1, 20% low), frequency of running out of food without having money to pay for more (4/13, 31%; n=2, 50% often; n=2, 50% sometimes; and n=0, 0% never), trouble paying for heat or electricity (5/13, 38%; n=1, 20% always; n=2, 40% sometimes; and n=2, 40% never), and likelihood of looking for a job in the near future (6/13, 46%; n=2, 33% high; n=3, 50% low; and n=1, 17% none).

##### Perceived Impact of Program on Motivation to Stop Smoking

All 12 participants who completed the questionnaires agreed or strongly agreed that participating in the program made them think about quitting smoking. Of the 12 participants, 8 (67%; n=4, 50% *ready to quit*, and n=4, 50% *not ready to quit*) agreed or strongly agreed with “The program made me think that it is okay to quit tobacco and other drugs at the same time”; 1 (8%) participant in the *ready to quit* track was undecided, and 3 (38%; n=2, 67% *ready to quit*, and n=1, 33% *not ready to quit*) disagreed. Participants perceived that the program increased their motivation to quit (overall mean 6.2, SD 1, range 1-7; *ready to quit* mean 6.7, SD 0.5; *not ready to quit* mean 5.4, SD 1; Table 2).

Of the 21 participants, 15 (71%) individuals who responded to at least one weekly SMS text message assessment on the helpfulness in motivating smoking cessation (1=not helpful to 5=very helpful) believed the program was helpful: 12 (response rate 57%; mean 3.6, SD 0.85) in week 1, a total of 10 (response rate 48%; mean 4, SD 0.9) in week 2, a total of 6 (response rate 29%; mean 4.3, SD 0.94) in week 3, and a total of 7 (response rate 33%; mean 4.4, SD 0.7) in week 4.

### Qualitative Data

Of the 21 participants, 13 (62%) participated in the 2- and 4-week interviews: 6 (46%) completed both, 4 (31%) completed only the 2-week interview, and 3 (23%) completed only the 4-week interview. Supporting quotes were identified by patient number, enrolled track (*ready to quit* or *not ready to quit*), and interview week (2 or 4 weeks).

#### Engagement and Interactions With the Text Messaging Program

Participants described that they frequently read the SMS text messages:

There were a couple of times I was in with a client or something—when I had a minute to read it, I would read it. It was never that I didn’t go back to it.

P3, ready to quit, 4 weeks

Approximately 15% (2/13) of participants had low response rates for the SMS text message assessments. When probed for the reasons, they responded as follows:

I responded to a couple of them and then didn’t respond anymore 'cause I didn’t know if it was being charged to my account.

P10, ready to quit, 4 weeks

I can’t read or write that much. I wait until my friend comes. When she comes, she reads them to me. Sometimes I got to wait two or three days.

P14, not ready to quit, 2 weeks

#### Program Satisfaction

Participants were satisfied with the program, largely because they found the SMS text messages understandable:

The thing I liked about the text messaging program is that it was straightforward. It wasn't hard for me to grasp the concept of what it was talking about.

P7, not ready to quit, 4 weeks

Several described how they liked being able to go back to look at the messages:

Sometimes I would go back and look and see if there was anything helpful that could help me at the time. It was helpful.

P10, ready to quit, 4 weeks

Features participants thought particularly helpful included the interactive features:

I didn’t think they were going to answer that quick. For them answering quick, it helped with my craving.

P1, ready to quit, 4 weeks

#### Perception of Program Content

##### Text Messages About Cost-Savings Associated With Stopping Smoking

Many described the cost-saving messages as helpful:

I used to buy two packs, and now I’d buy one pack, so I’d say I’m going to smoke one, and I’d put the rest of the money in the piggy bank. Since I started with you guys, I have $120 in my piggy bank.

P14, not ready to quit, 4 weeks

##### Text Messages About Provision of Resources for Unmet SDOH Needs

Some described how receiving links to resources was helpful:

Because it gave you all the information, where to call or how to get in contact with people to try to get help...they definitely helped me out, ‘cause at that time when the message came in I was low on canned food’.

P1, ready to quit, 4 weeks

Others preferred communicating directly with an advocate:

I would’ve liked that somebody get in touch with me and to advocate to help me finish housing.

P9, ready to quit, 4 weeks

##### Text Messages About Managing Stress

Participants found the SMS text message tips on handling stress helpful:

When I had the stress tips—proper breathing we do. Yeah, I found that helpful.

P1, ready to quit, 4 weeks

##### Text Message Framing Around the Theme: Patients Who Quit Smoking Have Higher Success in Quitting Other Substances

For some, the SMS text messages about how quitting cigarettes and opioids together could help them remain abstinent from all substances were particularly helpful:

I like the ones for quitting other things at the same time as smoking 'cause that seems really hard for me to do.

P4, not ready to quit, 4 weeks

Others did not find these messages relevant as they were already on medication-assisted treatment for OUD:

I didn’t need help for that (opioids). I just needed help for smoking.

P9, ready to quit, 4 weeks

##### Text Messages That Provide Resources About Smoking Cessation Services

SMS text messages regarding where to find smoking cessation resources were viewed as helpful*:*

It has nice little facts about smoking. You have a number that gets you on medication. And, once I tried medication, it helped me out.

P5, not ready to quit, 2 weeks

##### Perceived Impact of the Program on Motivation to Stop Smoking

Individuals indicated that the program was beneficial for motivating smoking cessation:

It was like your mother in your ear, reminding you of stuff. Not in a nagging way. I was kind of surprised that it worked as well as it did but happily so.

P3, ready to quit, 4 weeks

Participants described that a major reason for increased motivation to stop smoking was social support:

Sometime I was having cravings, and that moment I would receive those text message like it was telling me somebody’s out there. I’m not by myself with quitting smoking. It’s like I have a sponsor.

P21, ready to quit, 4 weeks

#### Suggestions for Improvement

Suggestions for improvement were (1) providing supportive phone calls when needed, (2) including personal success stories, and (3) including educational videos:

Maybe have live people to talk to when you crave something.

P5, not ready to quit, 2 weeks

...to have people that have already smoked and quit have some of their personal story incorporated.

P10, ready to quit, 4 weeks

I like to receive education video about quitting smoking.

P21, ready to quit, 4 weeks

## Discussion

### Principal Findings

Individuals with OUD meet the definition of an underserved population: they have a higher smoking prevalence than the general population, disproportionate burden of tobacco-related health disparities, increased risk factors for treatment failure, and lack of protective factors [41]. Tailored interventions for underserved populations are needed to avoid treatment failure for those seeking treatment, as well as to motivate those not ready to stop smoking. We provide evidence of the feasibility and acceptability of a newly developed SMS text messaging program for smoking cessation tailored to individuals with OUD.

Our SMS text messaging program is unique because it (1) focuses on an understudied and underserved population, (2) assesses and provides resources for unmet SDOH needs that may make the path to quitting easier, and (3) offers individualized tracks based on readiness to quit. The main findings were as follows: (1) participants reported high satisfaction with the program content and structure, and (2) participants reported that the program helped motivate smoking cessation. The vast majority of participants in our study were Medicaid insured. Medicaid recipients in Massachusetts (MassHealth members) have access to all Food and Drug Administration–approved medications, two 90-day treatment regimens per year, and 16 tobacco cessation counseling sessions per year; however, the quit rates are low. Our study adds to the growing body of evidence that SMS text messaging approaches are feasible and acceptable for providing tobacco treatment to all individuals who smoke, even among low-income Medicaid populations who have OUD and are not ready to quit.

Individuals reported that the program made them think about stopping smoking, regardless of whether they were enrolled in the *ready to quit within 30 days* or *not ready to quit within 30 days* track. SMS text messages on managing stress and providing tobacco treatment resources were perceived as particularly helpful. Many perceived that the SMS text messages provided social support, companionship, and the motivation to stop smoking. Messages about the health benefits of quitting were well received, whereas messages on how quitting cigarettes may prevent relapse from other substances had mixed views, highlighting the importance of tailoring interventions to patient preferences.

Participants made suggestions for improvement. Some discussed how increasing the duration to 3 months would enhance the program, as would receiving supportive calls or supplemental in-person interactions as needed. Although some perceived that providing links to resources for unmet SDOH needs was adequate, others suggested that an advocate should additionally help address these needs. As suggested by the data, we plan to refine the intervention by increasing the program duration and adding supplemental in-person interactions, particularly to address unmet SDOH needs.

### Comparison With Prior Work

A previous intervention using SmokefreeTXT (an SMS text messaging service by the National Cancer Institute) with individuals experiencing homelessness demonstrated a median response rate of 2.1% to interactive SMS text messages, with many individuals reporting that the SMS text messages felt impersonal [42]. Our SMS text messaging program tailored to unmet SDOH needs addresses the unique circumstances of this population. Response rates to SMS text message assessments in our study ranged from 30% to 42%, with participants perceiving the program as customized to their needs.

In another study that analyzed the completion of the SmokefreeTXT program, 46% of those who set a quit date remained enrolled for the entire 42-day program. Among users who did not complete the program (eg, texted “STOP”) before program completion, the mean number of days in the program was 12 days [43]. In our study, although none of the participants dropped out of the program (eg, texted “STOP”), only 45% completed the 4-week assessments, perhaps indicating that some of these individuals did not complete the entire program. Similar findings have also been reported in other SMS text messaging interventions in underserved populations, as well as in other understudied populations [42,44,45], such as women who smoke cigarettes, where >60% of participants did not answer their phones to conduct interviews, despite multiple attempts [44].

### Limitations

This study has several strengths and limitations. The strengths include focusing on the understudied and underserved population of individuals with OUD and including individuals regardless of readiness to quit. These inclusions are important as evidence supports “opt-out” approaches to offering tobacco treatment to all individuals, regardless of readiness to quit [46-52]. Many studies exclude individuals with substance use disorders or psychiatric diseases [47,48,53], thus perpetuating health inequities. For qualitative studies, participants were interviewed both during and immediately after the study completion, thus minimizing recollection bias. However, our small sample size from a single recruitment site limited generalizability. Our results also reflect the findings of participants who volunteered and thus may not reflect the perspectives of all individuals. Another limitation was that we found that a significant number of participants were ineligible during face-to-face screening as they did not have unlimited SMS text messaging or a cell phone during hospitalization. Future studies could provide cell phones or SMS text messaging plans to patients at discharge. Although our understudied population was a strength, it created a limitation for assessing feasibility and acceptability; we were unable to reach half of the participants by phone at the end of the study. This, coupled with limited resources, limited our ability to collect and measure smoking abstinence. Unlike other studies, we derived SMS text message engagement data from multiple sources (unsolicited SMS text messages, SMS text message response rates, self-report surveys, and interviews). In addition, most, if not all, SMS text message programs are limited by a lack of ability to ascertain whether the message was read. In future studies, we can additionally offer incentives to individuals responding to SMS text message assessments or make messages more interactive through the use of quizzes or questions.

### Conclusions

SMS text messaging to promote smoking cessation may be an effective tool for improving quit rates and health outcomes in individuals who smoke cigarettes and have OUD. Our results provide valuable insights into the development and acceptability of such programs. An innovative component of our SMS text messaging intervention was screening for and providing tailored resources for unmet SDOH needs. In future studies, we will assess whether identifying unmet SDOH needs and intervening in these modifiable factors (ie, providing resources to address unmet SDOH needs) affects smoking cessation. Our next steps are to further refine the program based on patient suggestions, such as adding a community health worker or coach to address unmet SDOH needs and providing supportive phone calls when needed, and assess the effects of the refined program on smoking cessation in a randomized controlled trial.

## Appendix

Multimedia Appendix 1 Schedule for delivering social determinants of health assessments.

## Abbreviations

BMC: Boston Medical Center
OUD: opioid use disorder
SDOH: social determinants of health
THRIVE: Tool for Health and Resilience In Vulnerable Environments
