# Supplementary figures and images for: Integrating Social Determinants of Health With Tobacco Treatment for Individuals With Opioid Use Disorder: Feasibility and Acceptability Study of Delivery Through Text Messaging
Source: JMIR Form Res. 2022 Sep 1;6(9):e36919. doi: 10.2196/36919 (PMC9478816; doi:10.2196/36919)

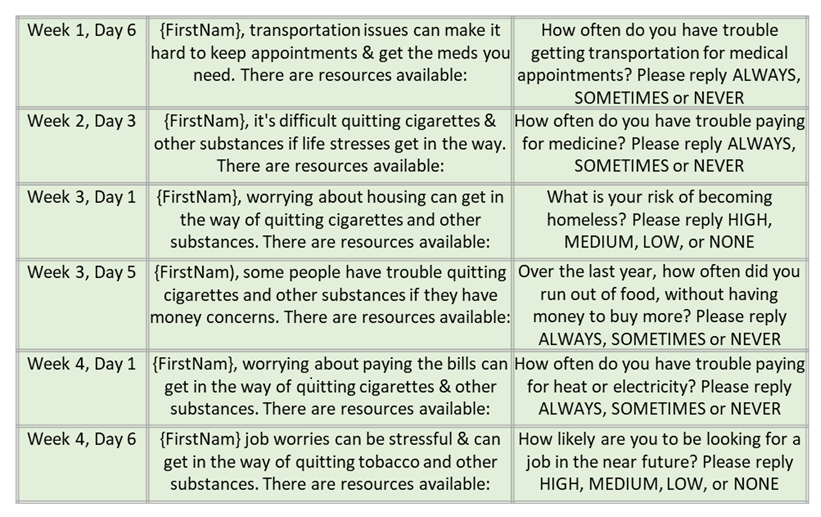

Supplement: Multimedia Appendix 1 [file formative_v6i9e36919_app1.png]
